# p38 MAP kinase-mediated NMDA receptor-dependent suppression of hippocampal hypersynchronicity in a mouse model of Alzheimer’s disease

**DOI:** 10.1186/s40478-014-0149-z

**Published:** 2014-10-21

**Authors:** Arne A Ittner, Amadeus Gladbach, Josefine Bertz, Lisa S Suh, Lars M Ittner

**Affiliations:** Dementia Research Unit, School of Medical Sciences, Faculty of Medicine, UNSW Australia, Sydney, Australia; Neuroscience Research Australia (NeuRA), Sydney, Australia

**Keywords:** Alzheimer’s disease, Amyloid beta precursor protein, Electroencephalography, NMDA receptor, Hypersynchronicity, p38 MAP kinase

## Abstract

Hypersynchronicity of neuronal brain circuits is a feature of Alzheimer’s disease (AD). Mouse models of AD expressing mutated forms of the amyloid-β precursor protein (APP), a central protein involved in AD pathology, show cortical hypersynchronicity. We studied hippocampal circuitry in APP23 transgenic mice using telemetric electroencephalography (EEG), at the age of onset of memory deficits. APP23 mice display spontaneous hypersynchronicity in the hippocampus including epileptiform spike trains. Furthermore, spectral contributions of hippocampal theta and gamma oscillations are compromised in APP23 mice, compared to non-transgenic controls. Using cross-frequency coupling analysis, we show that hippocampal gamma amplitude modulation by theta phase is markedly impaired in APP23 mice. Hippocampal hypersynchronicity and waveforms are differentially modulated by injection of riluzole and the non-competitive N-methyl-D-aspartate (NMDA) receptor inhibitor MK801, suggesting specific involvement of voltage-gated sodium channels and NMDA receptors in hypersynchronicity thresholds in APP23 mice. Furthermore, APP23 mice show marked activation of p38 mitogen-activated protein (MAP) kinase in hippocampus, and injection of MK801 but not riluzole reduces activation of p38 in the hippocampus. A p38 inhibitor induces hypersynchronicity in APP23 mice to a similar extent as MK801, thus supporting suppression of hypersynchronicity involves NMDA receptors-mediated p38 activity. In summary, we characterize components of hippocampal hypersynchronicity, waveform patterns and cross-frequency coupling in the APP23 mouse model by pharmacological modulation, furthering the understanding of epileptiform brain activity in AD.

## Introduction

Alzheimer’s disease (AD) is characterized by cognitive decline, and presents histopathologically with amyloid-β (Aβ) deposition in extracellular plaques and intracellular neurofibrillary tangles made up of hyperphosphorylated tau [1,2]. Besides these pathological hallmarks, it is becoming increasingly clear that AD is associated with alterations in neuronal circuit excitability. Patients with Alzheimer’s disease have an increased risk of developing neuronal hypersynchronicity, seizures or other forms of epileptiform activity [3,4]. AD is associated with a 5- to 10-fold increase in seizure incidence, and seizure incidence correlates with lower cognitive performance [4]. Although seizures were previously thought to be secondary to disease progression, aberrant neuronal activity may directly contribute to cognitive deficits, as neuronal activity appears to regulate regional vulnerability to Aβ [5]. Modulating epileptiform hippocampal activity modulates cognitive deficits in patients with mild cognitive impairment [6]. Conversely, forms of epilepsy are accompanied by cognitive impairments [7,8]. Thus, epileptiform activity may contribute to the development of AD-related cognitive deficits. However, the aetiology of epileptiform activity in AD is incompletely understood.

Synchronous network oscillations contribute to normal brain activity, yet, are affected by epileptogenesis [9,10]. In the hippocampus, two main oscillations reflect physiological synchronous activities, both being implicated in behavioural states and memory performance: theta oscillations (4-12 Hz), generated by synchronous phasic firing of pyramidal cells [11,12], and gamma oscillations (25-100 Hz), generated by circuits between GABAergic interneurons and pyramidal cells [13]. GABAergic interneurons play a key role in synchronization of pyramidal cell firing by providing rhythmic inhibition of pyramidal cells [14]. In addition, hippocampal interneurons are themselves synchronized by recurrent excitation [13]. Neuronal networks show further levels of interconnectivity: the phase of theta rhythms modulates the amplitude of gamma oscillations in the hippocampus, and this phase-amplitude cross-frequency coupling (CFC) is critical in hippocampus-dependent memory and cognitive performance [15-20]. Whether epileptiform activity in AD correlates with aberrations in CFC between hippocampal theta and gamma oscillations is not known. However, aberrant generation of electroencephalographic oscillations has been observed in cognitive impairment and AD [21], and aberrant hippocampal oscillations have been suggested to occur early in development of AD [22].

Neuronal expression of human amyloid-β precursor protein (APP) carrying pathogenic mutations has been used to model AD in mice [23]. APP transgenic mice exhibit extensive aberrant neuronal network and epileptiform activity [24-32]. Modulating hyperactivity and seizure susceptibility in the hippocampus modulates cognitive deficits in APP transgenic mice [27,31,33,34]. Hippocampal gamma spectral power, a measure of the contribution of gamma frequencies to the entire signal strength, is markedly diminished in 8 month-old behaving J20 mice expressing mutated hAPP [35]. Cross-frequency coupling between theta phase and fast gamma amplitude is altered in hippocampal slice cultures from APP transgenic mice [36]. However, a concise analysis of hypersynchronicity, spectral and CFC aberrations in adult APP mice *in vivo* is lacking. Furthermore, the pathways that contribute to network aberrations and hypersynchronicity in APP mice remain incompletely understood.

Oligomeric Aβ may itself affect neuronal circuit excitation [24]. Aβ reduces excitatory neuronal transmission and plasticity at the synaptic level [37-41]. Neuronal hyperexcitation and concomitant excitotoxicity in APP transgenic mice require the microtubule associated protein tau [2,33,42]. Dysfunction of synaptic NMDA receptors and their downstream signals was shown to underlie loss of inhibitory currents and abnormal hyperexcitation in hippocampal preparations from APP mice [31]. However, what contributions synaptic NMDA receptors and inhibitory neuron function have in generation and propagation of neuronal network aberrations and hypersynchronicity remains unclear. Furthermore, signalling pathways that may modulate thresholds for aberrant network activity are incompletely understood. Recently, mitogen-activated protein (MAP) kinase p38 has been implicated in the Aβ-induced inhibition of long-term potentiation (LTP) in brain slice cultures [43]. The role of p38 activity in neuronal network alterations of APP transgenic mice, however, has not been investigated.

In this study, we examined hippocampal hypersynchronicity in adult APP23 transgenic mice using *in vivo* telemetric electroencephalography (EEG) in free-roaming mice and analyse interictal recording sequences for spectral amplitude distribution and CFC strength before the onset of plaque pathology. We report spontaneous hippocampal hypersynchronicity in APP23 transgenic mice accompanied by marked spectral changes and impaired CFC for theta and gamma oscillations. Furthermore, we addressed thresholds of hypersynchronicity and interictal spectral and CFC distributions upon pharmacological manipulations of voltage-gated sodium channels, which regulate GABAergic inhibition, by riluzole and of NMDA receptors, by the non-competitive inhibitor MK801. Furthermore, we found that MK801 treatment significantly reduces activation of p38 MAP kinase in the hippocampus, and inhibition of p38 alters hippocampal hypersynchronicity thresholds in APP23 mice.

## Materials and methods

### Mice

APP23 transgenic mice on C57BL6 background were described previously [44]. All animal experiments were approved by the Animal Ethics Committee of the University of New South Wales. Mice were housed in 12 hour/12 hour light dark cycle with food ad libitum. Mice used in this study (electroencephalography and histology: 5 APP23 transgenic and 5 non-transgenic littermates; immunoblots: 3-4 mice per experimental group) were 4 month-old males.

### EEG implantation

Wire EEG electrodes on remote telemetric transmitters (DSI) were implanted as previously described [45]. Briefly, after anesthesia with ketamine/xylazine, scalp incision along the midline was performed. The head was fixed in a stereotactic frame (Kopf instruments) and the bregma was located. Bone openings were drilled using a bone micro-drill (Fine Science Tools, F.S.T.) at positions previously described for the hippocampus (x 2.0, y -2.0, z -2 with reference to bregma). Electrodes were inserted at this position with reference electrode placed above the cerebellum (x 0, y -6.0, z 0 from bregma). Electrodes were fixed in place by polyacrylate followed by wound closure and rehydration. Correct placement of electrodes was confirmed by serial sections of paraffin embedded brain tissue with hematoxylin-eosin staining. Only recordings from mice with proper placement of electrodes were included in further analysis. Two days after all EEG recordings were performed, animals were sacrificed by transcardial perfusion with cold phosphate-buffered saline (PBS) and brain samples were extracted for further processing for histological analysis.

### EEG data recording

Electroencephalograms were recorded with a DSI wireless receiver setup (DSI) with amplifier matrices using the Dataquest A.R.T. recording software at 500 Hz sampling rate [45]. Recordings were screened manually for movement artefacts and only artefact-free EEG passages were used in analysis. Raw LFP were noise filtered using a powerline noise filter (Neuroscore, DSI).

### EEG data analysis

Analysis of EEG recordings was performed using the NeuroScore software v3.0 (DSI) with integrated spike detection module. Spike trains were thus detected automatically and statistical data on spike train duration, frequency and number of spikes per train were obtained. Spectral analysis (i.e. analysis of signal power at individual frequencies expressed as square of the fast Fourier transform (FFT) magnitude) of intra-ictal sequences was performed using the integrated FFT spectral analysis function of NeuroScore. Frequency bands of theta and gamma wave forms were defined between 4-12 Hz and 25-100 Hz, respectively. Gamma and theta spectral contributions were quantified by area-under-curve (AUC) analysis across the defined frequency band in 3 artefact- and hypersynchronous spike-free sequences per recording (each 1 min in length). Cross-frequency coupling of theta phase and gamma amplitude was performed using MATLAB as previously described [20]. Briefly, for cross frequency coupling analysis, raw LFP was noise filtered using a powerline noise filter (Neuroscore, DSI). Noise-filtered LFP was filtered at two frequency ranges of interest for gamma (*f*_*A*_) and theta (*f*_*p*_). The phase time series for theta (*Φ*_*fp*_*(t)*) and the amplitude envelope time series for gamma (*A*_*fA*_*(t)*) were obtained by Hilbert transformation of the filtered LFPs. The combined series [*Φ*_*fp*_*(t), A*_*fA*_*(t)*] was then generated. After phase binning, the means *Ā*_*fA*_*(j)* of *A*_*fA*_ for each bin *j* were calculated and normalized using the sum ${\displaystyle {\sum}_{j=1}^N\overline{A}fA}(j)$ of *Ā*_*fA*_*(j)* over *N* bins to generate phase-amplitude distribution *P(j)*. The modulation index is based on calculating the Kullback-Leibler distance *D*_*KL*_ between the non-uniform (i.e. coupled) phase-amplitude distribution *P(j)* over all phase bins and the uniform (i.e. uncoupled) distribution *U(j)*.

$$ DKL\left(P,Q\right)={\displaystyle {\sum}_{j=1}^NP(j) log\left[\frac{P(j)}{U(j)}\right]} $$

The modulation index *MI* is defined as

$$ MI=\frac{DKL\left(P(j),U(j)\right)}{log(N)} $$

Phase-amplitude distributions and modulation indices were determined from artefact- and hypersynchronous spike-free 3 sequences (each 1 min) per recording before and after treatment.

### Antibodies

The following antibodies were used: phospho-p38 (Cell Signaling Technologies), p38 (Santa Cruz), APP (22C11), APP (6E10), glyceraldehydephosphate dehydrogenase (GAPDH) (all Millipore).

### Immunofluorescence

Immunostainings were performed as previously described [46]. Briefly, brain hemispheres from PBS-perfused mice were fixed with 4% paraformaldehyde in PBS at 4 degrees for 4 hours. Fixed brain samples were processed for paraffin embedding in a histoprocessor (Excelsior Tissue Processor, Thermo Shandon). Paraffin-embedded brain samples were sectioned (5 μm) on a microtome (Leica). After de-paraffinization, rehydration and washing, samples were incubated with blocking buffer (5%BSA, 1% horse serum, PBS pH7.4) and subsequently incubated with primary antibody overnight. Slides were washed and incubated with secondary antibody coupled to Alexa (Molecular probes) fluorophores in blocking buffer. After washing, samples were counterstained with 4′,6-diamidino-2-phenylindole (DAPI) and mounted with Fluoromount (Sigma-Aldrich, St. Louis, MO, USA) mounting medium.

### Western blotting

Brain tissue was extracted from mice immediately after cervical dislocation and brain hemispheres were dissected to isolate the hippocampus. Hippocampal isolates were homogenized immediately in RIPA buffer (50 mM Tris, pH 8, 150 mM NaCl, 1% Nonidet P-40, 5 mM EDTA, 0.5% sodium deoxycholate, 0.1% sodium dodecylsulfate (SDS), 0.02 mM NaF, 1 mM phenylmethylsulfonyl fluoride, 1 mM NaVO_4_ and protease inhibitors (Complete, Roche Applied Science)) by passing through a 29G syringe, incubated on ice for 20 min and centrifuged for 10 min at 12,000 × *g* and 4°C. Protein concentrations were determined by Bradford assay (BioRad). Western blotting was performed as previously described [47]. Equal amounts of protein samples were denatured in loading buffer (final concentrations: 62.5 mM Tris-HCl pH6.8, 2% SDS, 0.02% Bromophenol Blue, 1% β-mercaptoethanol) at 95°C for 5 minutes. Protein samples and molecular weight standards were loaded on 10% SDS-polyacrylamide gels and separated by electrophoresis. Proteins were transferred onto nitrocellulose membrane (Millipore) in transfer buffer (25 mM Tris, 192 mM glycine, 20% methanol, 0.1% SDS). Membranes were washed with TBST (10 mM Tris pH7.4, 150 mM NaCl, Tween-20 0.05%), blocked with 5% bovine serum albumin (Sigma-Aldrich) in TBST and probed with primary antibodies diluted in 5% bovine serum albumin (Sigma-Aldrich) in TBST overnight at 4°C or for 1 hour at room temperature. Primary antibodies used were: anti-p38 (Santa Cruz; 1:400), anti-phospho-p38 (Threonine180/Tyrosine182; Cell Signaling; 1:1000), anti-human APP (22C11; Chemicon; 1:1000), anti-GAPDH (Chemicon; 1:5000). After three washes with TBST, membranes were incubated with secondary antibody-HRP conjugate diluted in 5% bovine serum albumin (Sigma-Aldrich) in TBST. Secondary antibody horseradish peroxidase (HRP)-conjugates used were: goat-anti-rabbit conjugate (Santa Cruz, 1:5000), goat-anti-mouse conjugate (Santa Cruz, 1:5000). After three washes with TBST, HRP-catalysed enhanced chemoluminescence (ECL) reaction was visualized on a digital imager (ChemiDoc, BioRad).

### Statistics

Statistical analysis was performed using Graphpad Prizm. T-test was used for comparison of two sets of normally distributed data; ANOVA was used for comparison of more than 2 data sets.

## Results

### Spontaneous hippocampal spikes and spike trains in APP23 transgenic mice

**Figure 1:**
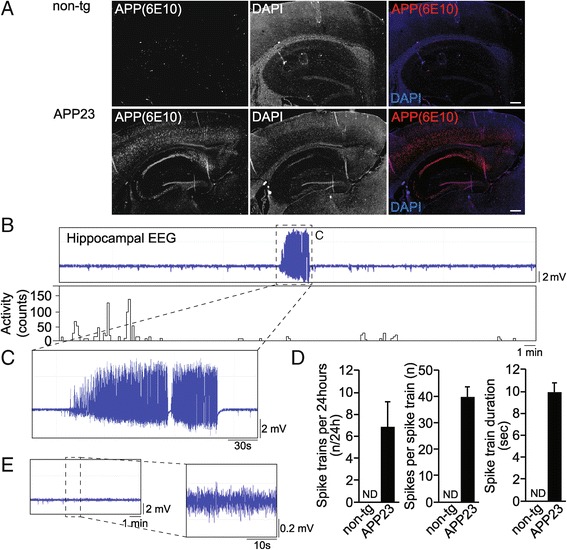
**Spontaneous hippocampal hypersynchronicity and spike trains in EEG of adult APP23 mice. (A)** Representative images of immunofluorescence stainings on hippocampal sections from non-transgenic (non-tg) and APP23 mice (4 months) for APP/Aβ (6E10). DAPI, 4′,6-diamidino-2-phenylindole. Scale bars, 100 μm **(B)** APP23 transgenic hippocampal EEG during active phase of day-night cycle. EEG trace (local field potential, LFP) (*upper panel*) and activity trace (*lower panel*) showing spontaneous spike activity and spike train (*marked up with dashed line*). Representative recording from 5 animals is shown. **(C)** Magnification of EEG trace in **B** showing representative spike train in hippocampal recording from APP23 transgenic mouse. **(D)** Spike train quantifications for APP23 and non-transgenic (non-tg) recordings over 24 hours. (n = 5) Values are mean ± s.e.m.; ND, none detected **(E)** Hippocampal EEG from non-transgenic mouse during active phase of day-night cycle. EEG trace and magnification of EEG trace (*marked up with dashed line*) are shown. Representative recording from 5 animals is shown.

To record hippocampal oscillations in APP23 transgenic mice, we implanted focal telemetric EEG electrodes into the hippocampus of male transgenic mice and non-transgenic littermates. APP23 mice at the age of study (4 months) showed prominent expression of APP and Aβ detected by the monoclonal antibody 6E10 in hippocampus and neocortex, however, in the absence of an overt amyloid plaque pathology (Figure 1A). Ten days after surgeries, EEG was recorded alongside activity for 24 hours. Frist, we analysed EEG traces from APP23 transgenic and non-transgenic mice for seizure spike trains, consisting of high-frequency polyspike or repetitive sharp wave structures. We found spontaneous spike trains in APP23 transgenic mice (Figure 1B,C and D; spike trains per 24 h: n = 5, *t* = 3.054, *p* = 0.0185 (non-transgenic vs APP23), Student t-test; spikes per spike train: n = 5, *t* = 2.221, *p* = 0.0346 (non-transgenic vs APP23), Student t-test; spike train duration: n = 5, *t* = 2.446, *p* = 0.0210 (non-transgenic vs APP23), Student t-test). Occurrence of spike trains did not correlate with increased locomotive activity, indicative of silent epileptiform episodes (Figure 1B). Hippocampal EEG recordings from non-transgenic littermates did not show spike activity (Figure 1E). Thus, APP23 transgenic mice display spontaneous epileptiform activity detectable by EEG.

### Altered spectral power of theta and gamma oscillations in APP23 mice

**Figure 2:**
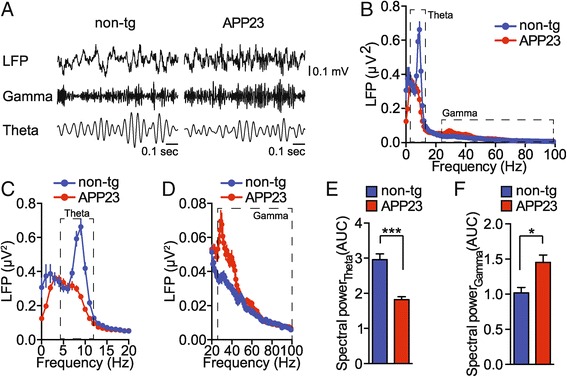
**Reduced theta and increased gamma spectral power in APP23 mice. (A)** Raw hippocampal EEG (LFP) and band pass filtered signals for theta (4-12 Hz) and gamma (25-100 Hz) oscillations in APP23 and non-transgenic (non-tg) mice. Representative signals from 5 animals per genotype are shown. **(B)** Spectral power of EEG waves in APP23 transgenic mice and non-transgenic (non-tg) controls during active phase. Dashed boxes mark theta (4-12 Hz) and gamma (25-100 Hz) oscillations. (n = 5) means ± s.e.m. **(C)** Magnified spectrum of theta oscillations. (n = 5) means ± s.e.m. **(D)** Magnified spectrum of gamma oscillations. (n = 5) means ± s.e.m. **(E)** Quantification of spectral power contribution of theta oscillations. (n = 5) means ± s.e.m. ***p < 0.001 (Student’s t-test) **(F)** Quantification of spectral power contribution of gamma oscillations. (n = 5) means ± s.e.m. *p < 0.05 (Student’s t-test).

Hippocampal theta (4-12 Hz) and gamma rhythms (25-100 Hz) are implicated in behavioural states and memory performance [11-13]. Theta and gamma rhythms have previously not been characterized in APP23 EEG recordings. To address the contribution of theta and gamma oscillations to the total power of recorded potentials, we performed spectral power analysis of interictal EEG recordings. APP23 mice showed markedly lower power of high frequency theta oscillations (~10 Hz) as compared with non-transgenic mice during the active phase of the light cycle (Figure 2A,B,C and E; n = 5 animals 6-8 measurements each, *t* = 7.029, *p* < 0.0001, Student’s t-test). In contrast, the spectral power of low frequency gamma oscillations (25-50 Hz) was markedly higher in APP23 mice as compared with non-transgenic controls (Figure 2A,B,D and F; n = 5 animals 6-8 measurements each, *t* = 2.569, *p* = 0.0116, Student’s t-test). Thus, APP23 transgenic mice present with inverse alterations in spectral power of hippocampal theta and gamma oscillations.

### Impaired hippocampal CFC in APP23 mice

**Figure 3:**
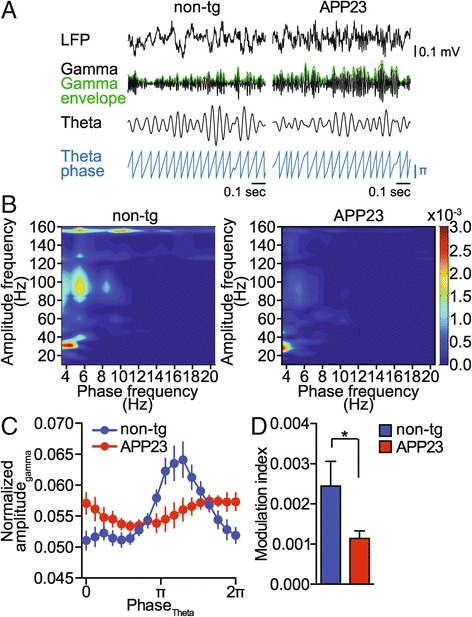
**Gamma amplitude modulation by theta phase is impaired in APP23 mice. (A)** Raw EEG (LFP), band pass filtered signals for theta (4-12 Hz) and gamma (25-100 Hz) oscillations, gamma amplitude envelope (green) and theta phase in APP23 and non-transgenic (non-tg) mice. Representative signals from 5 animals per genotype are shown. **(B)** Representative phase-amplitude comodulograms computed for hippocampal LFPs recorded in non-transgenic (non-tg) and APP23 mice. **(C)** Phase-amplitude plot computed for hippocampal LFPs recorded in non-transgenic (non-tg) and APP23 mice. (n = 5) means ± s.e.m. **(D)** Modulation index computed for the phase-amplitude distributions shown in **C**. (n = 5) means ± s.e.m. *p < 0.05 (Student’s t-test).

Hippocampal gamma and theta oscillations show marked CFC that is considered central to hippocampal cognitive functions such as spatial awareness and memory representation [16,48]. We addressed CFC in *in vivo* recordings from adult APP23 transgenic mice, which has not been done in APP expressing mice *in vivo* thus far. On noise-filtered interictal recordings, APP23 mice showed markedly fewer events of CFC coinciding with peaks of the gamma amplitude envelope and the theta phase, as compared with non-transgenic mice (Figure 3A). To address phase-amplitude coupling across theta (4-12 Hz) and gamma (25-100 Hz) frequencies, we used a comodulogram plot that simultaneously reports the level of coupling among multiple bands on the basis of scanning frequency band pairs [20]. Comodulogram plots of non-transgenic mice show several frequency pairs with significant coupling, in particular at ~6 Hz (Figure 3B). In contrast, APP23 comodulogram plots showed markedly lower coupling intensities across theta frequencies (Figure 3B). To quantify CFC in relation to theta phase, we plotted phase-amplitude distributions for 1-minute segments of hippocampal recordings for APP23 and non-transgenic controls during the active phase of the light cycle (Figure 3C). Non-transgenic phase-amplitude distributions peak in synchrony with theta phase – indicative of significant CFC (Figure 3C). However, phase-amplitude distributions in APP23 recordings did not show a significant peak during the theta phase (Figure 3C). The modulation index (MI) was described as a robust statistics of CFC [20]. APP23 recordings used to calculate phase-amplitude distributions had a significantly lower modulation index compared to recordings from non-transgenic mice (Figure 3D; n = 5 animals 2-3 measurements each, *t* = 2.456, *p* = 0.0239, Student’s t-test). Taken together, our CFC analyses show that APP23 have significantly weaker modulation of gamma amplitude by the theta phase.

### Riluzole-induced acute hippocampal hypersynchronicity in APP23 mice

**Figure 4:**
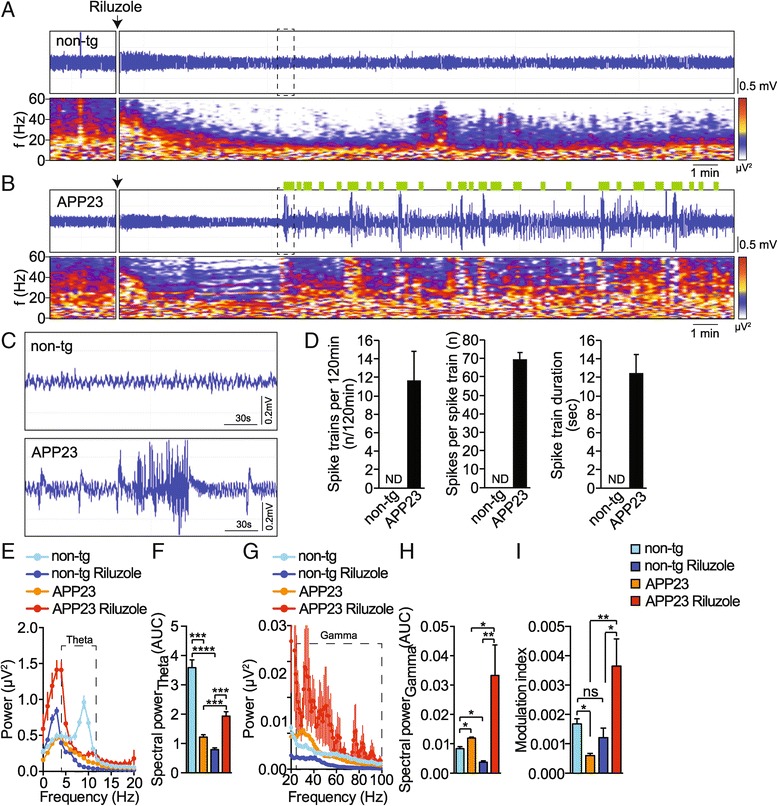
**Riluzole aggravates hippocampal hypersynchronicity in APP23 mice.** Representative EEG trace (LFP) and periodogram (0-60 Hz) of **(A)** non-transgenic and **(B)** APP23 transgenic mice, before and after administration of riluzole (20 mg/kg i.p.). **(C)** magnification of EEG traces marked in **A** an **B** by dashed boxes. (n = 5). **(D)** Spike train quantification for APP23 and non-transgenic (non-tg) recordings after injection with riluzole. (n = 5) Means ± s.e.m.; ND, none detected **(E)** Spectral power analysis of APP23 and non-transgenic (non-tg) recordings before and 20 minutes after injection of riluzole. Dashed box marks theta band. Means ± s.e.m. (n = 5). **(F)** Quantification of spectra in **E**. AUC, area under curve. Means ± s.e.m. ****p < 0.001, ***p < 0.005 (ANOVA) **(G)** Spectral power analysis of APP23 and non-transgenic (non-tg) recordings before and 20 minutes after injection of riluzole. Dashed box marks gamma band. Means ± s.e.m. (n = 5). **(H)** Quantification of spectra in **G**. AUC, area under curve. Means ± s.e.m. **p < 0.01 *p < 0.05 (ANOVA) **(I)** Modulation index before and 20 minutes after injection with riluzole. (n = 5) means ± s.e.m. **p < 0.01 *p < 0.05 ns, not significant (ANOVA).

Riluzole injection was shown to induce cortical hypersynchronicity in APP transgenic J20 mice [34]. However, the effects of riluzole on EEG in other APP transgenic mice as well as on additional EEG parameters (i.e. theta oscillations and CFC) remained to be shown. To test whether riluzole induces hippocampal hypersynchronicity in APP23 mice, we injected transgenic mice and non-transgenic controls intraperitoneally with riluzole and recorded hippocampal EEG before and after administration. In non-transgenic mice, riluzole induced a progressive and persisting reduction of oscillation amplitude and frequency (Figure 4A). In contrast, APP23 mice started showing increased and prolonged hypersynchronicity with repetitive spike activity and spike trains ten minutes after riluzole administration that remained throughout the recording period (Figure 4B,C and D; spike trains per 120 min: n = 5, *t* = 3.639, *p* = 0.0066 (non-transgenic vs APP23), Student t-test; spikes per spike train: n = 5, *t* = 2.884, *p* = 0.0063 (non-transgenic vs APP23), Student t-test; spike train duration: n = 5, *t* = 3.426, *p* = 0.0014 (non-transgenic vs APP23), Student t-test). Riluzole-induced spike trains in APP23 mice where interspersed by single spike events (Figure 4C). No spikes were detected in riluzole-treated non-transgenic mice. Riluzole had different effects on spectral periodograms recorded in non-transgenic and APP23 mice (Figure 4A,B). To quantify effects on spectral power of theta and gamma frequency bands, power spectra were averaged from recordings at 20 minutes after injection with riluzole and before treatment. At baseline, APP23 transgenic mice showed consistently and significantly weaker high-frequency theta waves (in particular those peaking at 10 Hz) compared to non-transgenic (Figure 4E,F; n = 5 animals 8-12 measurements each, *F*_3,181_ = 66.77, *p* < 0.0001, ANOVA). Upon injection with riluzole, theta power was markedly reduced in non-transgenic animals, however, significantly enhanced in APP23 transgenic mice, in particular at low frequencies (≤4 Hz) (Figure 4E,F). Power of gamma oscillations was higher in APP23 spectra compared to non-transgenic control recordings before riluzole injection (Figure 4G,H; n = 5 animals 8-12 measurements each, *F*_3,181_ = 7.141, *p* = 0.0001, ANOVA). While non-transgenic mice showed significantly suppressed gamma oscillations upon riluzole injection, APP23 spectra showed a significant relative increase of gamma oscillation power after injection with riluzole (Figure 4G, H). Riluzole had no significant effect on theta-gamma CFC in non-transgenic mice as indicated by similar modulation index values (Figure 4I; n = 5 animals 2-3 measurements each, *F*_3,38_ = 6.014, *p* = 0.0019, ANOVA). In contrast, APP23 recordings showed marked CFC impairment before riluzole injection, and a surprising increase in coupling strength was measured after riluzole injection (Figure 4I). Thus, marked modulation of oscillations at gamma and theta frequencies and an enhanced CFC strength are concomitant with increased hypersynchronicity upon injection with riluzole in APP23 mice compared to non-transgenic controls.

### Non-competitive NMDA receptor inhibition enhanced spike activity in APP23 transgenic mice

**Figure 5:**
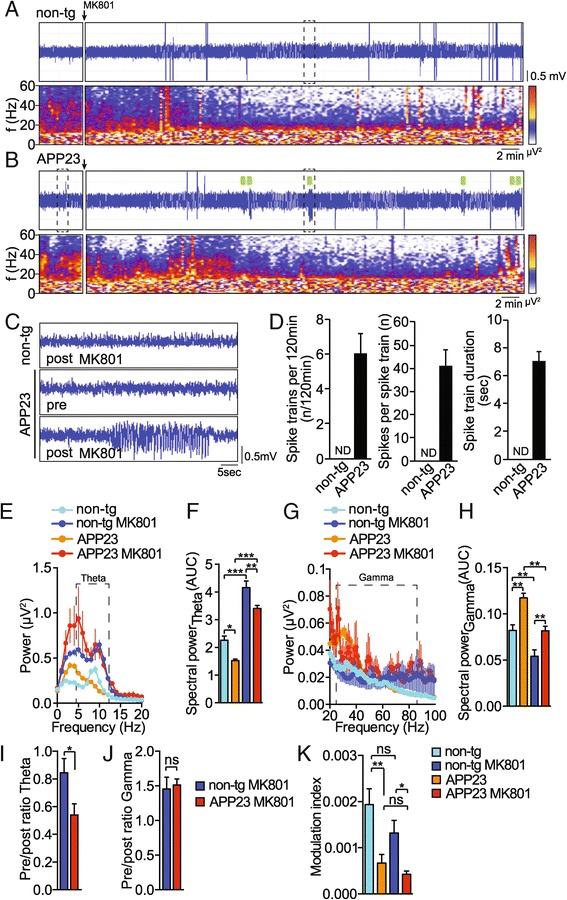
**NMDA receptor inhibition enhances hippocampal spike activity in APP23 mice.** Representative EEG trace (LFP) and periodogram (0-60 Hz) of **(A)** non-transgenic and **(B)** APP23 transgenic mice before and after injection of MK801 (0.4 mg/kg i.p.). **(C)** magnification of EEG traces marked in **A** an **B** by dashed box. (n = 5) **(D)** Spike train statistics for APP23 and non-transgenic (non-tg) recordings after injection of MK801. (n = 5) Mean ± s.e.m.; ND, none detected **(E)** Spectral power analysis of APP23 and non-transgenic (non-tg) recordings before and 20 minutes after injection of MK801. Dashed box marks theta band. Means ± s.e.m. (n = 5). **(F)** Quantification of spectra in **E**. AUC, area under curve. Means ± s.e.m. ****p < 0.001 ***p < 0.005 **p < 0.01 *p < 0.05 (ANOVA) **(G)** Spectral power of EEG waves in APP23 transgenic mice and non-transgenic (non-tg) controls before and 20 minutes after injection of MK801. Dashed box marks gamma band. Means ± s.e.m. (n = 5). **(H)** Quantification of spectra in **G**. AUC, area under curve. Means ± s.e.m. **p < 0.01 (ANOVA) **(I)** Ratio of theta power pre/post-injection of MK801. (n = 5) means ± s.e.m. * p < 0.05 (Student’s t-test) **(J)** Ratio of gamma power pre/post-injection of MK801. (n = 5) means ± s.e.m. not significant (Student’s t-test) **(K)** Modulation index before and 20 minutes after injection of MK801. (n = 5) means ± s.e.m. **p < 0.01 *p < 0.05 ns, not significant (ANOVA).

Direct inhibition of NMDA receptors may affect hypersynchronicity in APP23 mice. Therefore, we treated APP23 mice and non-transgenic controls with the non-competitive NMDA receptor inhibitor MK801, and recorded hippocampal EEGs before and after injection. Upon MK801 injection, non-transgenic mice showed no indication of hypersynchronicity (Figure 5A,C and D). In contrast, MK801 injection resulted in increased occurrence of hypersynchronicity in APP23 mice (Figure 5B, C and D; spike trains per 120 min: n = 5, *t* = 2.360, *p* = 0.0244 (non-transgenic vs APP23), Student t-test; spikes per spike train: n = 5, *t* = 2.364, *p* = 0.0244 (non-transgenic vs APP23), Student t-test; spike train duration: n = 5, *t* = 2.499, *p* = 0.0176 (non-transgenic vs APP23), Student t-test). Periodograms show MK801 treatment-induced power and frequency shifts in theta frequencies (Figure 5A and B). In-depth spectral analysis revealed that, similar to non-transgenic recordings, MK801 induced strong increases in power of theta oscillations in APP23 spectra (Figure 5E,F; n = 5 animals 8-12 measurements each, *F*_3,186_ = 67.80, *p* < 0.0001, ANOVA). Power of gamma oscillations was suppressed to similar extents by MK801 in APP23 and non-transgenic mice (Figure 5G,H; n = 5 animals 8-12 measurements each, *F*_3,186_ = 20.70, *p* < 0.0001, ANOVA). However, strength of gamma oscillations remained still higher in APP23 spectra compared with non-transgenic spectra after MK801 injections (Figure 5G,H). Calculation of the ratio of spectral power pre and post injection of MK801 in non-transgenic and APP23 recordings showed that MK801 had a significant effect on theta power ratio in APP23 mice (Figure 5I n = 5 animals 8-12 measurements each, *t* = 2.750, *p* = 0.0175, Student’s t-test), yet did not affect gamma power ratios (Figure 5J n = 5 animals 8-12 measurements each, *t* = 0.3210, *p* = 0.7491, Student’s t-test), suggesting a pronounced involvement of MK801-induced theta power changes correlating with MK801-induced hypersynchronicity. CFC impairments in APP23 mice persisted before and after MK801 injections and coupling strength was not affected by MK801 (Figure 5K; n = 5 animals 2-3 measurements each, *F*_3,23_ = 5.768, *p* = 0.0043, ANOVA). Taken together, while MK801 treatment enhances the power of theta waveforms in hippocampal EEG in APP23 and in non-transgenic mice, it does not affect CFC strength. However, MK801 induces hippocampal hypersynchronicity in APP23, suggesting that the threshold to hypersynchronicity in transgenic mice is governed by NMDA receptors.

### MK801 inhibits p38 MAP kinase activity in the APP23 hippocampus

**Figure 6:**
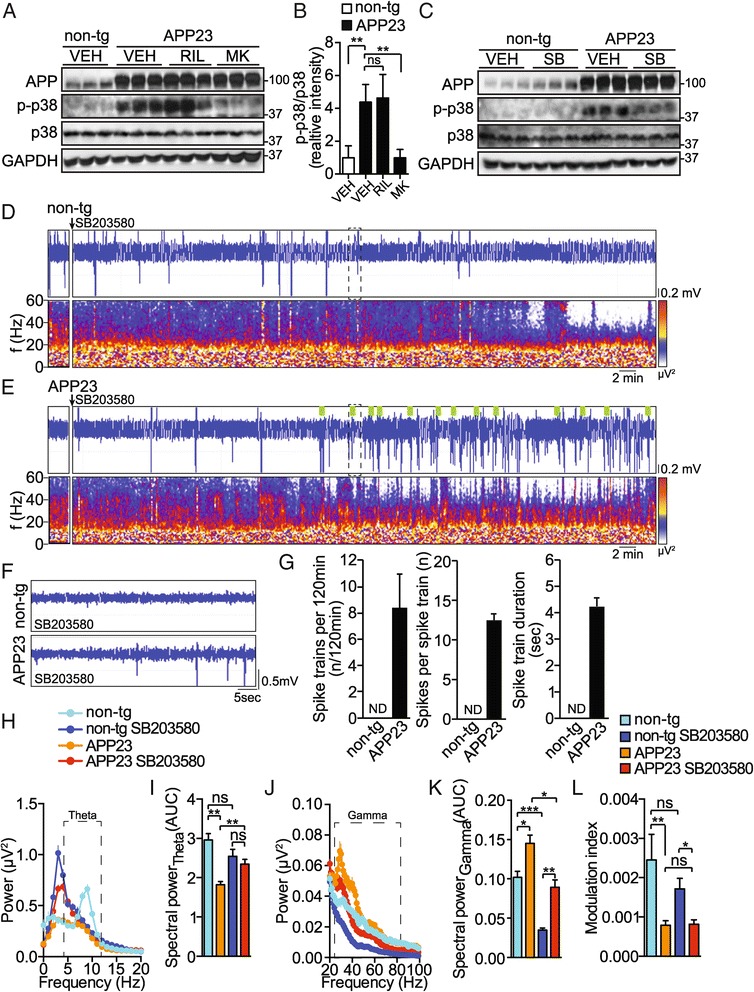
**The p38 inhibitor SB203580 enhances hippocampal spike activity in APP23 mice. (A)** Immunoblot using hippocampal lysates from APP23 transgenic or non-transgenic (non-tg) mice injected with riluzole (RIL), MK801 (MK) or vehicle control (VEH) probed for human APP, phospho-p38, p38 and GAPDH. Representative blot with 3 mice per group is shown. **(B)** Quantification of immunoblots (n = 3-4) means ± s.d. **p < 0.01 (ANOVA) **(C)** Immunoblot of hippocampal lysates from APP23 transgenic mice or non-transgenic (non-tg) injected with SB203580 (SB, 0.4 mg/kg i.p.) or vehicle control (VEH) probed for human APP, phospho-p38, p38 and GAPDH. Representative blot with 3 mice per group is shown. **(D, E)** Representative EEG trace (LFP) and periodogram (0-60 Hz) of **(D)** non-transgenic and **(E)** APP23 transgenic mice before and after injection of SB203580 (0.4 mg/kg i.p.). **(F)** Magnified EEG traces marked in **D** and **E** by dashed box. (n = 5). **(G)** Spike train quantification. (n = 5) Means ± s.e.m.; ND, none detected **(H)** Spectral power analysis of APP23 and non-transgenic (non-tg) recordings before and 60 minutes after injection of SB203580. Dashed box marks theta band. Means ± s.e.m. (n = 5). **(I)** Quantification of spectra in **H**. AUC, area under curve. Means ± s.e.m. **p < 0.01 ns, not significant (ANOVA) **(J)** Spectral power of EEG waves in APP23 transgenic mice and non-transgenic (non-tg) controls before and 60 minutes after injection of SB203580. Dashed box marks gamma band. Means ± s.e.m. (n = 5). **(K)** Quantification of spectra in **J**. AUC, area under curve. Means ± s.e.m. ***p < 0.005 **p < 0.01 *p < 0.05 (ANOVA) **(L)** Modulation index before and 60 minutes after injection of SB203580. (n = 5) means ± s.e.m. **p < 0.01 *p < 0.05 ns, not significant (ANOVA).

Pathways downstream of NMDA receptors in hippocampal neurons have been implicated in neurotoxicity, hypersynchronicity and pathology in AD mouse models including the APP23 transgenic mice [2,49]. Downstream events include calcium influx and activation of kinase signalling [49,50]. Aβ has been shown to regulate p38 MAPK downstream of NR2B-containing NMDA receptors [43]. Here, we investigated the effect of riluzole and MK801 on p38 activation (i.e. phosphorylated p38) in the hippocampus of APP23 mice using Western blotting. While riluzole did not significantly alter the levels of p38 phosphorylation, APP23 mice injected with MK801 showed markedly lower hippocampal p38 phosphorylation (Figure 6A,B). p38 activation has been reported at very early stages in AD [51] and in APP transgenic mice [52]. Accordingly, we found higher hippocampal levels of active p38 in APP23 mice compared to non-transgenic controls (Figure 6C). Furthermore, hippocampal phospho-p38 levels were sensitive to systemic application of p38 inhibitor SB203580 (Figure 6C). Thus, increased p38 activation in APP23 could be reduced by blocking NMDA receptors.

### p38 inhibition enhances hippocampal hypersynchronicity in APP23 mice

The NMDA receptor-dependence of p38 phosphorylation in APP23 mice prompted us to investigate the effects of the p38 inhibitor SB203580 on hippocampal EEG. SB203580 treatment did not induce hypersynchronicity in non-transgenic mice (Figure 6D,F and G), yet significantly enhanced hypersynchronicity in APP23 transgenic (Figure 6E,F and G; spike trains per 120 min: n = 5, *t* = 3.534, *p* = 0.0095 (non-transgenic vs APP23), Student t-test; spikes per spike train: n = 5, *t* = 3.063, *p* = 0.0032 (non-transgenic vs APP23), Student t-test; spike train duration: n = 5, *t* = 3.194, *p* = 0.0021 (non-transgenic vs APP23), Student t-test). Furthermore, SB203580-injected APP23 transgenic mice showed recurring individual spikes beginning at approximately 60 minutes post-injection (Figure 6F), followed by series of short spike trains (Figure 6E,G). SB203580 treatment resulted in a frequency shift of theta oscillations towards slow waveforms (peaking around 4 Hz) in both APP23 transgenic and non-transgenic mice non-transgenic mice (Figure 6H). However, quantitation of spectra over the entire theta band (4-12 Hz) showed no significant difference in non-transgenic mice before and after SB203580 treatment, yet an increase of overall power of theta oscillations in APP23 mice with SB203580 treatment (Figure 6I; n = 5 animals 8-14 measurements each, *F*_3,220_ = 12.28, *p* < 0.0001, ANOVA). SB203580 treatment led to significant decrease in gamma wave power in both APP23 and non-transgenic mice (Figure 6J,K; n = 5 animals 8-14 measurements each, *F*_3,220_ = 22.37, *p* < 0.0001, ANOVA). Similar to MK801 (Figure 5I), SB203580 treatment did not impact on interictal CFC strength in APP23 and non-transgenic mice (Figure 6L; n = 5 animals 3-4 measurements each, *F*_3,54_ = 7.212, *p* = 0.0004, ANOVA). Taken together, administration of the p38 inhibitor SB203580 induced enhanced hippocampal hypersynchronicity in APP23 mice, while modulating theta oscillations and suppressing gamma oscillations in APP23 and non-transgenic control mice.

## Discussion

In the present study, we report spontaneous hypersynchronicity in APP23 transgenic mice. Furthermore, we show that APP23 mice have marked alterations in theta and gamma oscillations and impaired cross-frequency gamma modulation by the phase of theta oscillations. We found markedly enhanced spike activity in APP23 after riluzole treatment. Surprisingly, we found that hypersynchronicity in APP23 mice could be enhanced by MK801 and the p38 inhibitor SB203580. Spectral analysis showed that enhanced hypersynchronicity in APP23 by riluzole, MK801 and SB203580 is accompanied by alterations in theta and gamma wave power. CFC analysis showed that impaired coupling strength in APP23 mice, while not altered by MK801 or SB203580 treatment, is significantly increased by riluzole (Table 1).

**Table 1:** **Summary of treatment effects on EEG measures in APP23 and non-transgenic controls**

	**Riluzole**		**MK801**		**SB203580**	
	**non-tg**	**APP23**	**non-tg**	**APP23**	**non-tg**	**APP23**
Hypersynchronicity	ND	**↑↑**	ND	**↑**	ND	**↑**
Spike trains per 2 hours (n/120 min)		11.6 ± 3.2		6.0 ± 1.2		8.3 ± 2.7
Spikes/spike train		69.4 ± 3.9		40.9 ± 7.0		12.4 ± 1.1
Spike train duration (s)		12.38 ± 2.09		6.97 ± 0.74		4.24 ± 0.35
Theta power	**↓**	**↑**	**↑**	**↑**	**→**	**→**
Theta frequency	**↓**	**↓**	**→**	**→**	**↓**	**→**
Gamma power	**↓**	**↑**	**↓**	**↓**	**↓**	**↓**
Cross-frequency coupling	**→**	**↑**	**→**	**→**	**→**	**→**
p38 MAPK activity	nt	**→**	nt	**↓**	**↓**	**↓**

**↑**, increase; **↓**, decrease; **→**, unchanged; ND, not detectable; nt, not tested. Values are means ± s.e.m.

Spontaneous hypersynchronicity, possible induced by Aβ [53], has previously been described for transgenic mouse models of AD [24-32,54]. Our study corroborates previous findings by showing spontaneous hypersynchronicity in APP23 mice, which express human APP carrying the Swedish double mutation. APP23 mice present memory deficits as early as 3 months of age [55], while plaque pathology is not seen before 6 months of age [44]. Our data show pronounced neuronal network aberrations and EEG abnormalities such as epileptiform activity, spectral power differences and CFC impairments in APP23 mice already at an age when memory deficits manifest, and well before Aβ plaque formation. This is paralleled by altered molecular signalling with hippocampal p38 activation. Neuronal networks may even be compromised before memory deficits develop, since network aberrations and hypersynchronicity have been observed in brain slices of APP-expressing TgCRND8 mice at 1 month of age [36,54].

Spectral analysis showed significantly lower interictal theta power in APP23 EEG recordings, while strength of gamma oscillations was significantly increased (Figure 2). As outlined above, hippocampal oscillations of theta and gamma frequencies contribute to physiological processes required in memory and cognition [11-13]. Thus, hippocampal network aberrations leading to spectral changes in these frequency bands may underlie onset of cognitive deficits in APP23 mice [55,56]. Alternative APP-expressing mouse models show similar alterations in theta and gamma waveforms [34,36,54]. Consistent with these findings in murine models, early human AD pathology and mild cognitive impairment is accompanied by alterations in neuronal network oscillations [21,22,24]. Whether altered spectral power of theta oscillations also influence thresholds for epileptiform activity in AD remains unclear. However, data from rodent models of epilepsy suggest that a slow oscillation state affects hypersynchronicity thresholds [57]. Thus, spectral power changes in theta oscillations may affect hypersynchronicity in APP23 mice.

Hippocampal gamma oscillations are generated by circuits between GABAergic interneurons and pyramidal cells [13,35]. Interestingly, our interictal hippocampal recordings show increased gamma oscillations in APP23 mice compared to recordings from non-transgenic mice (Figure 2). These findings suggest altered network topology in APP23 limbic systems, which potentially include aberrant circuit activity of GABAergic interneurons and pyramidal cells. In human epilepsy and rodent models of epilepsy, pre-seizure states are characterized by enhanced gamma oscillations [58]. However, these findings may not be directly translatable to epileptogenesis in APP transgenic mice. APP-expressing mice have been reported with changes in distribution and survival of GABAergic interneurons at early stages [59-61]. Cortical hypersynchronicity in APP mice and its relation to gamma oscillations and function of GABAergic interneurons has recently been investigated in detail [34]. Verret *et al.* elegantly showed that cortical gamma oscillations are impaired during strong hypersynchronicity in mutant APP transgenic J20 mice, compared to pre-ictal sequences within the same recordings. Different from this previous study, we focused on analysis of recording sequences that were not characterized by hypersynchronicity and made comparisons between APP23 and non-transgenic mice rather than of pre-ictal and ictal states. Thus, we do not conclude on gamma oscillations during hypersynchronicity states in the hippocampus of APP23 mice. Nevertheless, the study by Verret *et al.*, our study and findings by others [36,54] indicate that altered network oscillations are an inherent phenotype of APP transgenic mice.

To our knowledge, our study represents the first report of hippocampal CFC deficits in adult APP transgenic mice. Previously, slice recordings have been analysed for theta phase to gamma amplitude coupling, however, only very young mice were used [36]. Intriguingly, CFC and spectral changes were present before high levels of Aβ were detectable in these mice [36]. These findings and our data suggest that CFC deficits occur early and persist over time in APP transgenic mice. They may thus contribute to hypersynchronicity and behavioural/memory deficits in APP transgenic mouse models that are already seen at this age. However, until now there are no mechanistic implications of CFC changes in epileptiform activity. So our study provides the first correlation of CFC deficits and hypersynchronous activity in the brains of APP-expressing mice. The above-mentioned study by Goutagny *et al* also describes alterations in spectral contributions from theta and gamma oscillations in slices of APP transgenic TgCRND8 mice [36], similar to what we detected in APP23 EEG recordings. CFC calculation measures used in our study (i.e. comodulogram plots, phase-amplitude distribution and modulation index) are based on normalized gamma amplitudes [20]. Therefore, these measures allow for quantitative coupling strength comparison between APP23 and non-transgenic recordings.

Riluzole blocks persistent sodium currents by inhibiting voltage-gated sodium channels [62]. *In vivo*, riluzole has anticonvulsive and sedative effects in rodents and humans [63,64]. We found that riluzole injection in APP23 mice leads to strong induction of hypersynchronicity, an increase in theta oscillation power, yet a shift towards lower theta frequencies. It also enhanced the power of gamma oscillations. A synchronized hippocampal slow oscillation state lowers the threshold for propagation and generation of epileptiform activity [57]. We found that riluzole causes frequency shift towards slow oscillations (≤4 Hz) in interictal EEG sequences, thus potentially instigating a slow oscillation state that lowers hypersynchronicity thresholds. This is neither seen with MK801 nor with SB203580, where interictal recordings still show a significant component of theta (4-12 Hz) in APP23 mice and induced hypersynchronicity was less pronounced (Table 1). Thus, the oscillatory state of hippocampal slow waves may be deterministic of seizure thresholds in APP transgenic mice.

Riluzole has been shown to enhance hypersynchronous states in cortical recordings from APP-expressing J20 mice [34]. Interestingly, riluzole suppressed gamma oscillations in cortical recordings from J20 mice [34], which is consistent with its effect on hippocampal gamma oscillations in APP23 mice in our study. We extended our analysis of riluzole-induced changes in EEG parameter to CFC between theta and gamma oscillations. Surprisingly, CFC strength was markedly increased in APP23 mice after riluzole injection, even though these mice show CFC impairment before injection. Even though non-transgenic mice show reductions in theta and gamma power, the effects of riluzole-injection on CFC in non-transgenic mice are insignificant, suggesting that riluzole has unique effects in APP-expressing mice that might impact directly or indirectly on generation and/or propagation of hypersynchronous activity. Our data are the first to correlate an increase in CFC strength with enhanced hypersynchronicity in EEG recordings in mice. The close correlation of riluzole-induced interictal surge in CFC strength and increased epileptiform activity in APP23 mice may imply a causal connection on a network level. Thus, our data using the APP23 mouse model tentatively imply that CFC analysis algorithms may prove useful to predict epileptiform episode in recordings of AD patients.

Voltage-gated sodium channels, targeted by riluzole, show altered expression and processing in mouse models of AD, which correlates with aberrant EEG activity and memory impairments, as well as altered sensitivity to riluzole [34,60]. The contribution of riluzole-sensitive sodium channels in cortical interneurons has been suggested to underlie the hypersynchronous effect of riluzole on cortical neuron networks [34]. This may to some extent translate to the hippocampal network in APP23 mice, as function of hippocampal GABAergic interneurons is impaired in epilepsy [14]. In the rodent hippocampus, expression of riluzole-sensitive channels is not restricted to interneurons, but also occurs in pyramidal CA neurons [65,66]. Thus, inhibition of voltage-gated sodium channels may have broad impact on neuronal networks in the hippocampus and – as secondary effect – may cause suppression of glutamate release [63,67]. How, such broad sodium channel inhibition contributes mechanistically to hippocampal hypersynchronicity remains unclear. Interestingly, riluzole had opposite effects on theta and gamma spectral power in our recordings from non-transgenic and APP23 mice. This suggests that riluzole induces a specific interictal state in APP mice that combines increased low frequency theta, enhanced gamma oscillations and a pronounced gamma-theta coupling that might lower network thresholds for epileptogenesis. Given the direct mode of action of riluzole is inhibition of voltage-gated sodium channels, the dissimilarity of EEG responses of non-transgenic and APP23 mice is likely due to differences in expression of these channels as shown for other APP-expressing mice [34].

Next, we used MK801, a non-competitive activity-dependent inhibitor of NMDA receptors, to address effects of NMDA receptor inhibition on hypersynchronicity and other EEG parameters in APP23 mice. Surprisingly, MK801 induced significant epileptiform activity in hippocampal recordings from APP23 mice, albeit to a lower extent as seen after administration of riluzole. Interestingly and different from the effects of riluzole, MK801 injection resulted in strongly enhanced theta oscillation and suppressed gamma oscillations, whilst not leading to frequency shifts (Table 1). MK801 also had significant effects of on theta and gamma spectral power in non-transgenic recordings. Therefore, we calculated the ratios of spectral power pre- and post-injection with MK801, which showed that theta, but not gamma, spectral power was significantly affected in APP23 mice. Furthermore, MK801 injection did not affect CFC strength. Thus, we argue that MK801 instigates a markedly different oscillatory state – in particular of theta rhythms - than riluzole in the hippocampus of APP23 mice, though both induce forms of hypersynchronicity. This suggests that a component in hypersynchronicity thresholds of APP23 mice is governed by NMDA receptors. Furthermore, neuronal populations may contribute differentially to hypersynchronicity in APP23 mice. In epilepsy models, MK801 inhibits epileptogenesis [68] or has no effects [69]. Therefore, the APP-dependent network alterations leading to hypersynchronicity may be unique to AD models with compromised NMDA receptor function. Interestingly, *stargazer* mice with a mutation in the *Cacng2* gene that encodes stargazin, a protein that regulates AMPA receptor trafficking and excitability in interneurons, show increased seizure length and hyperexcitability upon NMDA receptor inhibition by MK801 [70]. AMPA receptor trafficking may be affected in APP transgenic mice. Consistent with this idea, Aβ and expression of mutated APP have been shown to reduce synaptic prevalence of AMPA receptors, thereby affecting synapse function [40,71].

We found p38 MAP kinase activity to be increased in APP23 mice. Interestingly, MK801, but not riluzole treatment results in lowered p38 MAP kinase activation in the hippocampus of APP23 mice (Figure 6A, Table 1). Surprisingly, suppressing hippocampal p38 activity with a pyridylimidazole-based inhibitor results in increased hypersynchronicity. Hence, increased p38 activity in APP23 hippocampus may be part of a protective mechanism limiting synchronized activity of neuronal circuits. MK801 treatment of APP23 mice resulted in similarly enhanced hypersynchronicity as after p38 inhibitor administration. Even though p38 inhibition – despite effective in the hippocampus – may affect other brain areas or organ systems (e.g. peripheral nervous system, vascular system), the effects seen with MK801 or p38 inhibitor could be mechanistically linked within hippocampal neurons. In support of this, NMDA receptors have been linked to p38 activation during promoting neuronal death [43,49,72]. Interestingly, Aβ or mutated APP expression increases activation of p38 [43,52]. Consistent with this, p38 might govern hypersynchronicity in APP mice downstream of MK801-sensitive NMDA receptors and this pathway may constitute a protective signal from epileptiform activity in this AD model. The molecular details of p38 signalling in this context as well as effects of long-term suppression of p38 activation on network aberrations and epileptiform activity remain to be investigated. Whether long-term inhibition of p38 is achievable in mice without affecting normal brain or peripheral function is, however, unclear, as longitudinal inhibitor studies of these functions are not available at present.

In summary, hippocampal EEG recordings in adult 4-month old APP23 mice show spontaneous hypersynchronicity, alterations in theta and gamma oscillation and impaired coupling of theta phase and gamma amplitude. Although, we found no apparent plaque burden in the 4 months-old APP23 mice analysed here, APP23 mice show memory deficits as early as 3 months of age and increased Aβ levels, as shown by others and us before [42,44,55]. While we determined EEG alterations in APP23 mice at a single age (4 months), other APP-expressing strains show hypersynchronicity and network aberrations at earlier and later stages, suggesting that APP23 mice likely show similar changes at similar ages [34,36,54]. Furthermore, differentially enhanced components of hypersynchronicity in APP23 mice after treatment with either riluzole, MK801 or SB203580, suggest involvement of sodium channels, NMDA receptors and p38 MAP kinase signalling in governing thresholds of hypersynchronicity in AD mouse models. The profound effects of short-term treatments with riluzole, MK801 or SB203580 on hippocampal EEG may well translate into changes in behaviour and memory performance specifically in APP23. Whether they are also reflected by changes in distribution or survival of neuronal subtypes (e.g. GABAergic interneurons) in the hippocampus of APP23 mice remains to be determined.

## Abbreviations

NMDA: N-methyl-D-aspartate
GABA: Gamma-aminobutyric acid
Hz: Hertz
